# Strengthening local health systems and governance for Universal Health Coverage: experiences and lessons from the COVID-19 pandemic response in Quezon City, Philippines

**DOI:** 10.1093/heapol/czaf002

**Published:** 2025-01-13

**Authors:** Esperanza Anita Escano-Arias, Ramona Asuncion D. G Abarquez, Rolando V Cruz, Rosalie Espeleta, Madeline Mae Ong, Arianna Maever Loreche, Veincent Christian F Pepito, Vida Gomez, Manuel M Dayrit

**Affiliations:** Local Government of Quezon City, Quezon City 1100, Philippines; Local Government of Quezon City, Quezon City 1100, Philippines; Local Government of Quezon City, Quezon City 1100, Philippines; National Capital Regional Office, Department of Health, Mandaluyong City 1550, Philippines; School of Medicine and Public Health, Ateneo de Manila University, Pasig City 1604, Philippines; School of Medicine and Public Health, Ateneo de Manila University, Pasig City 1604, Philippines; National Clinical Trials and Translation Center, National Institutes of Health, University of the Philippines Manila, Pedro Gil St., Ermita, Manila 1000, Philippines; Department of Epidemiology and Biostatistics, University of the Philippines Manila, Pedro Gil St., Ermita, Manila 1000, Philippines; School of Medicine and Public Health, Ateneo de Manila University, Pasig City 1604, Philippines; World Bank Philippines Country Office, 5th Ave cor 25th St, Bonifacio Global City, Taguig 1634, Philippines; School of Medicine and Public Health, Ateneo de Manila University, Pasig City 1604, Philippines

**Keywords:** local government, health systems strengthening, Universal Health Coverage, COVID-19 pandemic, pandemic preparedness, devolution, health security

## Abstract

The COVID-19 pandemic has disrupted the Philippines’s transition toward universal health coverage. However, some local government units in the country made use of the pandemic as a catalyst to strengthen their local health system, scale-up the provision of preventive and primary care services, and improve health governance to make it more prepared to face future pandemics and realize the aims of the country’s new Universal Healthcare Act. This paper describes the response of the local government of Quezon City, Philippines, to COVID-19 and how it strengthened local health systems. We also discuss enablers such as partnerships, collaborations, and foresight to ensure that investments during the pandemic will continue to be of use. We also identify some constraints and propose recommendations to consolidate local health system gains during the COVID-19 pandemic response in the transition toward universal health coverage.

Key messages

- The disruption caused by the COVID-19 pandemic led the Quezon City local government in the Philippines to mount a response that strengthened its local health system and its capacity to implement the country’s Universal Healthcare Act (Republic Act 11 223) of 2019.
- Investments during the COVID-19 pandemic should be sustained for pandemic preparedness and health systems strengthening during the transition towards Universal Health Coverage.
- Universal Healthcare Act impact measures such as out-of-pocket spending and quality of care should be estimated annually at the local government level through the Local Government Scorecard on Health to measure progress in attaining Universal Health Coverage.

## Introduction

The COVID-19 pandemic has exposed health system vulnerabilities globally (Pradana et al. 2020, Dutta and Fischer 2021, Talabis et al. 2021) and has further disrupted progress towards achieving Universal Health Coverage (UHC) (Amit et al. 2021, 2022, Talabis et al. 2021). Despite (or because of) this, local governments have made significant efforts to improve health systems and governance (Yates et al. 2024). However, there are few studies that report on health system strengthening experiences and challenges during the COVID-19 pandemic. In this paper, we document how the local government unit of Quezon City, Philippines used the COVID-19 pandemic as an impetus to scale-up the provision of health services and to strengthen the responsiveness and adaptability of its health system. By documenting the experience of Quezon City, we present a model that countries with similar health systems and resources may learn from in moving towards a UHC- and pandemic-ready health system.

### One of the Philippines’s largest cities

Quezon City is the Philippines’s most populous city with 3.2 million people as of 2022. Since 2016, the city is the most competitive in the country, scoring high on health infrastructure, disaster risk reduction plan, sanitary system, and early warning system (Department of Trade and Industry 2023). As of 2021, the average annual income of families in the city is PHP 429 100 (US$7300) in current prices (Philippine Statistics Authority 2022). With a revenue of PHP 23.37 billion (US$412.2 million) in 2020 alone, the city is one of the country’s most economically important (Commission on Audit (Philippines) 2021). On the other hand, it is also home to some of the most densely packed residential areas in the country. The city’s average population density is 17 000/km^2^ as of 2020 (Philippine Statistics Authority 2020). As of 2018, the poverty incidence in Quezon City is 1.5%. For the year 2021, the top three causes of death are COVID-19, with 5963 deaths, acute myocardial infarction, with 5700 deaths, and pneumonia with 1747 deaths (Philippine Statistics Authority 2023).

The private sector, the national government, and the local government run many tertiary or specialty hospitals in the city. Due to devolution (Dayrit et al. 2018, Department of Health (Philippines) 2019), local health systems development is largely the responsibility of local government through the Quezon City Health Department (QCHD).

## Implementation of COVID-19 response strategies while strengthening the health system

A consequence of the Philippines’s devolved health system is that the national government designs the overall COVID-19 response strategy of the country, but the local governments are responsible for its operationalization and implementation. The Philippine government’s strategy towards COVID-19 was prevent, diagnose, isolate, treat, reintegrate, and vaccinate (PDITR + V), which was operationalized by QCHD through lockdowns and quarantine, monitoring, testing and contact tracing, disease surveillance, and vaccination (Department of Health (Philippines) 2022a). The effects of operationalizing this strategy for each of the six building blocks of health are described as follows (Table 1).

**Table 1.** Innovations in COVID-19 response and post-pandemic residuals to strengthen Quezon City’s health system for each of the health system’s building blocks

Health system’s building blocks	Innovations in COVID-19 response	Post-pandemic residuals
Health leadership and governance	1. Tasking and clarifying roles and responsibilities in implementing national government strategy Development of ordinances (supplementary Table 1) and executive orders 2. Collaboration and partnership building 3. Community mobilization 4. Establishment of facilities a. Molecular laboratory b. Isolation and quarantine facilities c. COVID-19 wards in hospitals run by the local government	1. Establishment of super health centers for each district in the city. 2. Ordinances enacted to establish an independent Health Education and Promotion Unit (HEPU) and upgrade the QCESU to a Division 3. Improving operational structure 4. Maintaining partnerships and collaborations
Human resources for health (HRH)	1. Hiring of additional HRH to help mitigate the effects of the pandemic 2. Additional HRH through collaboration and community mobilization 3. All additional HRH trained on disease surveillance	Hired additional HRH are integrated in the QCHD structure
Health financing	1. Utilization of emergency funds from local government 2. Increase in the budget to respond to the emergency	Increase in QCHD budget from PHP 771 624 444 (USD 13.76 million) or 4.06% in 2018 to PHP 2 299 042 670 (USD 41.00 million or 5.89% of total local government budget) in 2024
Health technology	1. Establishment of molecular laboratory 2. Establishment of cold storage facility for COVID-19 vaccines	1. Molecular laboratory now upgraded to public health laboratory to diagnose other diseases. 2. Cold-storage facility is now used to house vaccines for National Immunization Program and is being expanded.
Health information	1. Tanod Kontra Covid for contact tracing 2. BantAI COVID System	1. TKC integrated into disease surveillance 2. BantAI COVID System developed into Bantay Kalusugan 3. Quezon City Health Information System (HIS) developed
Service delivery	1. Special concern lockdown—local government provides for the essentials of families to ensure adherence to quarantine protocols 2. Free community-based testing a. Prioritization b. Industry-wide active surveillance 3. Provision of basic services including food and medicine 4. Telemedicine and teleconsultation 5. Use of social media platforms for health education and promotion	1. Social media platform for health education integrated into HEPU 2. Telemedicine and consultation maintained 3. Community-based testing integrated into all health centers

### Health leadership and governance

Quezon City and the entire island of Luzon were put on lockdown in 2020 (Amit et al. 2021). The first step in operationalizing the response of Quezon City was tasking and delineating of roles of the different divisions and units within the local government. Many ordinances and executive orders were enacted and implemented (Supplementary Table 1, see online supplementary material). Community mobilization was essential in distributing aid from the City’s Social Services Development Department to quarantined and unemployed individuals. The prolonged lockdown eventually proved unsustainable and impractical as it deprived people of the opportunity to earn and the economy ground to a halt (Raza et al. 2022). To allow people to tend to their livelihoods and yet still control the infection, Quezon City implemented—among the first local government units in the country to do so—‘Special Concern Areas for Lockdown’ (SCAL). Instead of locking down large geographic areas and barangays (i.e. villages), SCAL allowed for isolation of smaller areas only where COVID-19 cases were concentrated (Mateo and Cayabyab 2020). This eventually became the model for future lockdowns implemented throughout the country, as targeted lockdowns mitigated the spread of the disease while minimizing gross domestic product losses by at least 20% (Del Castillo et al. 2024).

The pandemic required a whole of government response and even non-health related departments played crucial roles in the pandemic response: (i) the Business Permit and Licensing Department helped ensure that COVID-19 safety protocols were implemented by business establishments; while (ii) the Tourism Department identified and monitored quarantine facilities.

Even as COVID-19 was effectively put under control, the local government wanted to build on their health systems strengthening gains by establishing a super health center in every district of the city (22nd Quezon City Council 2023a). The operational structure of QCHD was also improved, with the city’s epidemiology and surveillance unit (QCESU) currently being transformed into a division with more resources and wider reach (22nd Quezon City Council 2023b). Similarly, the city’s health education and promotion unit is being made independent from formerly being a part of QCESU (22nd Quezon City Council 2023c) .

### Human resources for health

Before the pandemic, the QCESU only had nine employees; however, the pandemic resulted in the hiring of more than 2000 phone-based and face-to-face contact tracers, who were funded by the national and the local government. These newly-hired individuals were trained on contact tracing, disease surveillance, and data management. This increase in contact-tracing capacity allowed for the referral of close contacts to community-based sites where they could be tested for COVID-19 (Dayrit 2022). Around 200 (10%) of these trained contact tracers are still working as disease surveillance officers in health centers throughout the city today.

### Health financing

**Figure 1.**
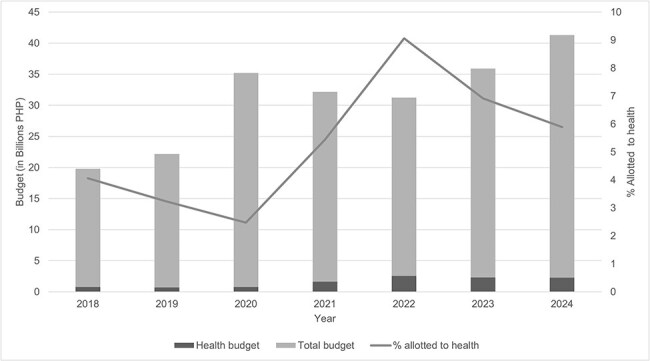
Budget of Quezon City Health Department in relation to the local government’s total budget, 2018–2024 (Local Government of Quezon City 2018, 2019, 2020, 2021, 2022, 2023, 2024).

In 2020, the budget of QCHD was only PHP 847 million (US$14.4 million), which is only 2.47% of the total local government’s budget (Fig. 1). The onset of the pandemic made this insufficient, thus emergency funds were deployed from the local government to help fund budgetary shortfalls. By 2021, the health budget was doubled to PHP 1.66 billion (US$28.8 million), and has stayed above PHP 2.00 billion (US$34 million) since 2022 to account for organizational reforms and sustained investments in health technology and infrastructure (Local Government of Quezon City 2018, 2019, 2020, 2021, 2022, 2023, 2024). In August 2024, QCHD received PHP 239 million (US$4.10 million) from the Philippine Health Insurance Corporation to support the city government’s implementation of medical services for their local constituents (Philippine Health Insurance Corporation 2024).

### Health information

During the onset of the COVID-19 pandemic, the Quezon City health information system had the following weaknesses: (i) a paper-based surveillance system that was cumbersome and error-prone (Junio 2021); (ii) delay in identifying patients who will receive medicine; and (iii) delay in identifying people who need to be contact traced as well as those who need to receive aid.

To address the first issue, QCESU transitioned to a web-based platform through the help of a civil society group called AI4Gov which co-developed and designed Tanod Kontra COVID (TKC) (AI4GOV 2023). TKC, which has been running since May 2021, shortened surveillance and contact-tracing turnaround times from 24 h to 1–4 h. Data collected from the ground can be encoded into TKC anywhere, which hastened the issuance of quarantine and isolation orders for confirmed cases and their close contacts. At present, TKC has been expanded for use for other respiratory illnesses similar to COVID-19.

To address the other issues, the Quezon City Identification System (QCID) was created (Local Government of Quezon City). Those who receive aid and those who should have check-ups in health centers preferably should be part of the QCID. Building on the QCID, the Quezon City Health Information System (QCHIS) is an electronic medical record currently being developed by a third-party provider with funding from the local government. After completion, it will be integrated among health centers first, and then made interoperable with other tertiary hospitals to allow for real-time disease surveillance, reporting, and action.

### Availability of medicines, vaccines, health technologies, and service delivery

The volume of COVID-19 RT-PCR confirmatory tests eventually proved expensive and tedious. To mitigate this problem, the local government entered into agreements with the different private molecular laboratories. Eventually, however, the city decided to build its own molecular laboratory, with the machines being donated by the private sector, and cartridges, capital outlay, and human resources costs being shouldered by the local government. This new laboratory not only increased the city’s testing capacity, but also reduced costs and waiting time. At present, the molecular laboratory is currently being upgraded to become capable of diagnosing other diseases (e.g. hepatitis, human immunodeficiency virus (HIV), human papilloma virus, tumor markers, etc.) so that it can continue to be of service beyond the pandemic (Mateo 2020).

To bridge human resources and technological gaps, the city used the BantAI COVID program, which is an artificial intelligence-powered system used for contact tracing, monitoring, and telemedicine referrals (Nievera 2022), that ensured continuity of care (Noceda et al. 2023). At present, the BantAI COVID system may be employed as a telemedicine platform to be used in COVID-19 and other diseases such as HIV.

Because non-essential travel was forbidden and classes were conducted online during the pandemic, hotels, public schools, and universities run by the local government were transformed into isolation and quarantine facilities. In these facilities, medicines and daily supplies of food and other necessities were provided by the local government. Severe and critical COVID-19 cases, however, were treated in the city’s specialist hospitals.

When the COVID-19 vaccines started coming in, there was no facility that could store the volume of vaccines needed to meet the city’s vaccination targets. Initially, there was a partnership with a pharmaceutical logistics company to handle the cold chain from storage to the vaccination sites; however, this proved expensive. With technical assistance from the United Nations Children's Fund, the city was able to build its own cold-storage facility. Utilizing pop-up vaccination sites, night vaccination for day workers, and vaccination programs in homes, restricted institutions, and jails, the city was rapidly able to vaccinate 2.17 million of its adult population from March 2021 to June 2022, far exceeding its target of 1.8 million people (Escano-Arias and Eleria 2022). At present, the city’s cold-storage facility is being used to store vaccines for the country’s National Immunization Program and plans are underway to expand the current facility.

## Achievements

**Figure 2.**
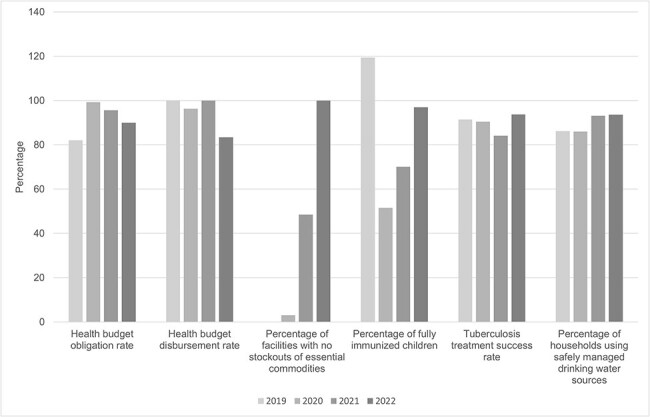
Performance on select indicators based on the local government unit scorecard on health for Quezon City, 2019–22 (Department of Health (Philippines) 2020, Department of Health (Philippines) 2021, Department of Health (Philippines) 2022b).

The health system quickly evolved to respond to the public health crisis, which is reflected in its consistently high health-budget obligation and disbursement rates, tuberculosis treatment success rates, and percentage of households with safe drinking water sources. The percentage of fully immunized children has reached 97% of the target after the initial slump due to the pandemic. The percentage of facilities with no stockouts of essential medicines and supplies rose from 0% in 2019 to 100% in 2022 (Fig. 2), partly due to investments in cold-storage facilities and procurement reform (Department of Health (Philippines) 2020, Department of Health (Philippines) 2021, Department of Health (Philippines) 2022b).

## Enablers

The city’s health-strengthening initiatives during the pandemic have been successful owing to intersectoral partnerships with national government and the private sector for building and supplementing infrastructure and resources; with the academe for understanding disease patterns and development of interventions (Sumpaico-Tanchanco et al. 2022, Caldwell, 2021); and with health workers and volunteers in the frontlines. Political will, commitment, and foresight were vital to ensure that resources invested during the pandemic are used for health systems strengthening (Escano-Arias and Eleria 2022).

## Challenges and constraints

There is a lack of proper monitoring on how well inputs in the different building blocks of health have translated to better utilization of quality health services at lower cost, which is the goal of the country’s Universal Healthcare Act (Bautista et al. 2022). This prevents local governments from accurately situating themselves and improving in their journey towards UHC. For example, out-of-pocket expenditures are not an indicator in the Local Government Scorecard on Health, despite it being an impact measure of UHC. Another vital UHC impact measure that is not being monitored is quality of care (Loreche et al. 2023), resulting in the perception that public health facilities have poor quality of care with their patients having poor satisfaction (Cagayan et al. 2020, Ulep et al. 2021). There had also been some difficulty in repurposing services developed during the COVID-19 pandemic as demand for them declined. For example, laboratories with RT-PCR purpose-built to diagnose COVID-19 were left idle and have not been repurposed yet, pending guidelines from the Department of Health, which should be done soon to prevent wastage.

## Conclusions

The COVID-19 pandemic made health a priority for national and local governments worldwide, which led to substantial investments (Yates et al. 2024). These investments should be sustained and repurposed to enable their use in other emerging health threats and in improving routine health services to further hasten the transition towards UHC. Investments made by the local government of Quezon City in diagnostic capacity, vaccine storage, and health information systems during the COVID-19 pandemic were repurposed for routine health services such as diagnoses of other diseases and storage of routine childhood vaccines, which led to improvements in vaccination rates and other health indicators. However, the journey towards UHC can further be hastened if impact measures such as out-of-pocket expenditures and quality of care can be monitored to assess progress. There should also be guidelines on how to repurpose services that arose during the COVID-19 pandemic (e.g., molecular diagnostic laboratories) towards strengthening molecular surveillance and diagnosis of other conditions to avoid wastage, as done in other settings (King et al. 2023). Through collaborations and partnerships, foresight, political will, and commitment, challenges were overcome by Quezon City to create a stronger health system that is more ready for UHC and for future health security threats.

## Supplementary Material

czaf002_Supp

## Reflexivity statement

E.A.E.A., R.A.D.G.A., and R.V.C. are employees of the Quezon City Health Department with more than 20 years each of experience in the design and implementation of health programs in an urbanized local government. R.E. is affiliated with the Department of Health National Capital Region Center for Health Development with more than 10 years of experience in the design and implementation of health policies at the regional level. M.M.O., A.M.L., and V.C.F.P. are researchers in the fields of medicine, health policy, and public health. V.G. is a senior health financing specialist with experience in the assessments of health financing strategies and systems in the Philippines and Myanmar. M.M.D. is a health systems researcher and former policymaker. He is a former Secretary of Health of the Republic of the Philippines and Director of the Department of Human Resources for Health at the World Health Organization. All researchers are based in the Philippines, except for MMO who is based in Australia.
