# Supplementary material for: Strengthening local health systems and governance for Universal Health Coverage: experiences and lessons from the COVID-19 pandemic response in Quezon City, Philippines
Source: Health Policy Plan. 2025 Jan 13;40(3):436–42. doi: 10.1093/heapol/czaf002 (PMC11886787; doi:10.1093/heapol/czaf002)
Supplement: czaf002_Supp [file czaf002_supp.zip › QC Paper_Supplementary Data.docx]

**Supplementary Data**

Supplementary Table 1. Select COVID-19 related ordinances passed by the Quezon City local government.

| Number | Ordinance | Date of implementation | Reference |
| --- | --- | --- | --- |
| SP-3125, S-2022 | An Ordinance amending subparagraph B (3) of paragraph A and subparagraph B (3) of paragraph B, both under section 3 of Ordinance No. 3114, S-2022, entitled “AN ORDINANCE REVISING ORDINANCE NO. SP-2273, S-2014, ENTITLED AND ORDINANCE ADOPTING THE SEAL OF GOOD HOUSEKEEPING IN ALL BARANGAYS OF QUEZON CITY, CONSOLIDATING THE AMENDMENTS PROVIDED IN ORDINANCE NOS. SP-2523, S-2016, SP-2588, S-2017, AND SP-2898, S-2020 AND INCORPORATING INDICATORS FOR COVID-19 RESPONSE.” | July 4, 2022 | (21st Quezon City Council, 2022a) |
| SP-3114, S-2022 | An Ordinance revising Ordinance No. SP-2273, S-2014, entitled “AN ORDINANCE ADOPTING THE SEAL OF GOOD HOUSEKEEPING IN ALL BARANGAYS OF QUEZON CITY,” consolidating amendments provided in Ordinance Nos. SP-2523, S-2016, SP-2588, S-2017, and SP-2898, S-2020 and incorporating indicators for COVID-19 response. | April 1, 2022 | (21st Quezon City Council, 2022b) |
| SP-3105, S-2022 | An Ordinance amending Ordinance No. SP-2987, S-2020, entitled “AN ORDINANCE MANDATING STRICT OBSERVANCE OF MINIMUM HEALTH PROTOCOLS BY PUBLIC TRANSPORTATION DURING THE COVID-19 PANDEMIC” in light of the declaration of Alert Level 1 in the National Capital Region | March 16, 2022 | (21st Quezon City Council, 2022c) |
| SP-3102, S-2022 | An Ordinance suspending the implementation of Ordinance No. SP-2985, S-2020, entitled “AN ORDINANCE PROVIDING FOR A SPECIAL PROTECTION OF CHILDREN AGAINST CORONAVIRUS DISEASE 2019 (COVID-19) BY SETTING CHILDREN PROTECTION HOURS WITHIN THE TERRITORIAL JURISDICTION OF QUEZON CITY, PROVIDING PENALTIES FOR VILOATION THEREOF, AND FOR OTHER PURPOSES” in light of the declaration of Alert Level 1 in Metro Manila, and granting authority to the City Mayor to restore the enforcement of the same in accordance with the national government policies and health exigency | March 15, 2022 | (21st Quezon City Council, 2022d) |
| SP-3101, S-2022 | An Ordinance suspending the implementation of Ordinance No. SP-2907, S-2020, as amended by Ordinance No. SP-3080, S-2022, entitled “AN ORDINANCE ESTABLISHING PROTOCOLS FOR THE MANAGEMENT OF THE DECEASED DURING THE COVID-19 PANDEMIC” | March 15, 2022 | (21st Quezon City Council, 2022e) |
| SP-3089, S-2022 | An Ordinance Suspending Ordinance No. Sp-3076, S-2022, Entitled “An Ordinance regulating the mobility of individuals unvaccinated for Covid-19 In Quezon City in light of the declaration of alert level 2 in Metro Manila, and granting authority to the City Mayor to restore the enforcement of the same in accordance with The National Government issuances. | February 7, 2022 | (21st Quezon City Council, 2022f) |
| SP-3077, S-2022 | An Ordinance amending Ordinance No. SP-3029, S-2021, entitled “AN ORDINANCE GRANTING FINANCIAL ASSISTANCE TO ALL FRONTLINERS WHO ARE TESTED POSITIVE TO CORONAVIRUS DISEASE 2019 (COVID-19) AND APPROPRIATING FUNDS THEREFOR”, to limit the Ten Thousand Pesos (PHP10,000.00) benefit only to cases requiring hospitalization. | January 12, 2022 | (21st Quezon City Council, 2022g) |
| SP-3076, S-2022 | An Ordinance regulating the mobility of individuals unvaccinated for COVID-19 in Quezon City | January 6, 2022 | (21st Quezon City Council, 2022h) |
| SP-3080, S-2022 | An Ordinance amending Ordinance No. SP-2907, S-2020, entitled “AN ORDINANCE ESTABLISHING PROTOCOLS FOR THE MANAGEMENT OF THE DECEASED DURING THE COVID-19 PANDEMIC” to allow limited wakes or burol | January 6, 2022 | (21st Quezon City Council, 2022i) |
| SP-3078, S-2022 | An Ordinance amending Ordinance No. SP-2958, S-2020, entitled “AN ORDINANCE ESTABLISHING INDIVIDUAL QUARANTINE PROTOCOLS DURING THE COVID-19 PANDEMIC”, as amended by Ordinance No. SP-3034, S-2021. | January 6, 2022 | (21st Quezon City Council, 2022j) |
| SP-3082, S-2021 | An Ordinance suspending the imposition of penalties, interests and surcharges to late payments of Tricycle Franchise Renewal during Community Quarantine only due to COVID-19 outbreak, subject to existing laws, rules and regulations. | December 29, 2021 | (21st Quezon City Council, 2021a) |
| SP-3061, S-2021 | An Ordinance amending Ordinance No. SP-2905, S-2020, entitled “AN ORDINANCE SETTING PUBLIC SAFETY HOURS WITHIN THE TERRITORIAL JURISDICTION OF QUEZON CITY FROM 8:00PM TO 5:00AM DUE TO THE CORONAVIRUS DISEASE 2019 (COVID-19) PANDEMIC, PROVIDING PENALTIES FOR VIOLATION THEREOF, AND OR OTHER PURPOSES”, for the purpose of suspending the enforcement of the same and giving authority to the City Mayor to reimpose the same when necessary to respond to a surge in COVID-19 cases. | November 15, 2021 | (21st Quezon City Council, 2021b) |
| SP-3048, S-2021 | An Ordinance Regulating Covid-19 vaccination sites and programs in Quezon City | September 2, 2021 | (21st Quezon City Council, 2021c) |
| SP-3032, S-2021 | An ordinance prohibiting the unauthorized or fraudulent sale, distribution or administration of COVID-19 vaccination cards, and other frauds in relation to COVID-19 vaccination and providing penalties for violation thereof and for other purposes | July 29, 2021 | (21st Quezon City Council, 2021d) |
| SP-3029, S-2021 | An ordinance granting financial assistance to all frontliners who are tested positive to CORONAVIRUS DISEASE 2019 (COVID-19), and appropriating funds therefor. | June 28, 2021 | (21st Quezon City Council, 2021e) |
| SP-3031, S-2021 | An ordinance prohibiting the selling and buying of Government Procured COVID-19 Vaccine and selling and buying of slots to avail of COVID Vaccination and providing penalties for violation thereof | June 16, 2021 | (21st Quezon City Council, 2021f) |
| SP-3024, S-2021 | An ordinance granting daily allowance to the COVID-19 Vaccination Volunteer Medical Staff. | June 1, 2021 | (21st Quezon City Council, 2021g) |
| SP-3014, S-2021 | An ordinance giving authority to the City Mayor to issue Emergency Measures to regulate the sale, distribution, and consumption of Liquor and other intoxicating beverages within the city, enforceable during the period of Public Health Emergency due to the COVID-19 pandemic, subject to the Inter-Agency Task Force for the Management of Emerging Infectious Diseases (IATF-MEID) and Metro Manila Council Guidelines. | March 31, 2021 | (21st Quezon City Council, 2021h) |
| SP-3003, S-2021 | An ordinance further amending Ordinance No. SP-2905, S-2020, entitled “An Ordinance Setting Public Safety Hours within the Territorial Jurisdiction of Quezon City from 8:00 P.M. to 5:00 A.M. due to the CORONAVIRUS DISEASE 2019 (COVID-19) Pandemic, providing penalties for violation thereof, and for other purposes,” by setting a new Public Safety Hours commencing from 12:00 A.M. to 4:00 A.M. | March 8, 2021 | (21st Quezon City Council, 2021i) |
| SP-2987, S-2020 | An ordinance mandating strict observance of minimum health protocols by Public Transportation during the COVID-19 Pandemic | December 16, 2020 | (21st Quezon City Council, 2020a) |
| SP-2985, S-2020 | An ordinance providing for a Special Protection of Children against CORONAVIRUS DISEASE 2019 (COVID-19) by setting children protection hours within the territorial jurisdiction of Quezon City, providing penalties for violation thereof, and for other purposes | December 15, 2020 | (21st Quezon City Council, 2020b) |
| SP-2983, S-2020 | An ordinance prohibiting the indiscriminate and improper disposal of face masks and other COVID-19 protective gears and products in all places within the territorial jurisdiction of Quezon City and prescribing penalties for any violation thereof | November 5, 2020 | (21st Quezon City Council, 2020c) |
| SP-2966, S-2020 | An ordinance approving Supplemental Budget Number 5, General Fund for fiscal year 2020, in the amount of One Billion One Hundred Fifty-five Million Five Thousand and Eight Hundred One Pesos (PHP1,155,005,801.00), to fund various maintenance and other operating expenses, property, plant and equipment expenditures in response to COVID-19 pandemic and for other purposes | October 7, 2020 | (21st Quezon City Council, 2020d) |
| SP-2958, S-2020 | An ordinance establishing individual quarantine protocols during the COVID-19 pandemic. | August 11, 2020 | (21st Quezon City Council, 2020e) |
| SP-2959, S-2020 | An ordinance providing guidelines for special concern lockdown to contain the COVID-19 pandemic. | August 3, 2020 | (21st Quezon City Council, 2020f) |
| SP-2950, S-2020 | An ordinance imposing a Kalinga Fare for all tricycles and pedicab-for-hire in Quezon City during the community quarantine due to COVID-19 pandemic | July 13, 2020 | (21st Quezon City Council, 2020g) |
| SP-2947, S-2020 | An ordinance amending Ordinance No. SP-2905, S-2020 entitled “AN ORDINANCE SETTING PUBLIC SAFETY HOURS WITHIN THE TERRITORIAL JURISDICTION OF QUEZON CITY FROM 8:00 P.M. TO 5:00 A.M. DUE TO THE CORONAVIRUS DISEASE 2019 (COVID-19) PANDEMIC, PROVIDING PENALTIES FOR VIOLATION THEREOF, AND FOR OTHER PURPOSES”, by setting an new public safety hours commencing from 10:00 PM to 5:00 AM | June 25, 2020 | (21st Quezon City Council, 2020h) |
| SP-2943, S-2020 | An ordinance enabling COVID-19 testing of select at-risk Quezon City Barangay Frontliners and Barangay Health Workers who, in the course of performing their duties, may have prolonged close contact with a suspect, confirmed or probable COVID-19 case within the jurisdiction of Quezon City | June 24, 2020 | (21st Quezon City Council, 2020i) |
| SP-2935, S-2020 | An ordinance requiring the mandatory COVID-19 testing for all Persons Deprived of Liberty (PDL) before admission to the Quezon City Jail and other similar circumstances | May 26, 2020 | (21st Quezon City Council, 2020j) |
| SP-2926, S-2020 | An Ordinance Granting Hazard Pay Of Two Hundred Pesos (PPH200.00) Per Day To All Barangay Personnel Who Physically Reported For Work In The Implementation Of The Enhanced Community Quarantine (ECQ) In Their Barangay Relative To The Covid-19 Pandemic. | May 11, 2020 | (21st Quezon City Council, 2020k) |
| SP-2922, S-2020 | An ordinance approving the Supplemental Budget No. 3, General Fund for Fiscal Year 2020, in the amount of One Billion and Four Hundred Thirty Million Pesos (PHP1,430,000.00) to fund various maintenance and other operating expenses in response to COVID-19 pandemic and for other purposes | April 30, 2020 | (21st Quezon City Council, 2020l) |
| SP-2923, S-2020 | An ordinance granting hazard pay to the Quezon City Government Personnel who physically report for work during the period of implementation of Enhanced Community Quarantine Relative to the COVID-19 pandemic | April 30, 2020 | (21st Quezon City Council, 2020m) |
| SP-2924, S-2020 | An ordinance granting of Special Risk Allowance to the frontline Quezon City Public Health Workers during the period of Enhanced Community Quarantine relative to the COVID-19 pandemic | April 30, 2020 | (21st Quezon City Council, 2020n) |
| SP-2909, S-2020 | An ordinance approving the Supplemental Budget No. 1, General Fund for the Fiscal Year 2020, in the amount of Two Billion and Eight Hundred Million Pesos (PHP2,800,000.00), in response to COVID-19 pandemic and for other operational expenses | April 16, 2020 | (21st Quezon City Council, 2020o) |
| SP-2911, S-2020 | An ordinance prohibiting any person, whether natural or juridical, from committing any act or forms of discrimination against any Corona Virus (COVID-19) infected persons, recovered patients, persons under monitoring (PUMs) or persons under investigation (PUIs), and against public and private doctors, nurses, health workers, emergency personnel and volunteers, and other service workers, who are assigned in hospitals and/or other health care institutions / centers where COVID-19 patients are being treated situated within the territorial jurisdiction of Quezon City, and providing penalties for violations therefor | April 16, 2020 | (21st Quezon City Council, 2020p) |
| SP-2912, S-2020 | An ordinance granting Financial Assistance to all frontliners who tested positive to Corana Virus Disease 2019 (COVID-19), and appropriating funds therefor | April 16, 2020 | (21st Quezon City Council, 2020q) |
| SP-2907, S-2020 | An ordinance establishing protocols for the management of the deceased during the COVID-19 pandemic | April 14, 2020 | (21st Quezon City Council, 2020r) |
| SP-2905, S-2020 | An ordinance setting public safety hours within the territorial jurisdiction Quezon City from 8:00PM to 5:00AM, due to the Corona Virus Disease 2019 (COVID-19) pandemic, providing penalties for violation therefor, and for other purposes | March 16, 2020 | (21st Quezon City Council, 2020s) |

**References**

21st Quezon City Council. 2020a. SP-2987, S-2020:An Ordinance Mandating Strict Observance Of Minimum Health Protocols By Public Transportation During The COVID-19 Pandemic. *Quezon City Government*.

21st Quezon City Council. 2020b. SP-2985, S-2020:An Ordinance Providing For A Special Protection Of Children Against CORONAVIRUS DISEASE 2019 (COVID-19) By Setting Children Protection Hours Within The Territorial Jurisdiction Of Quezon City, Providing Penalties For Violation Thereof, And For Other Purposes. *Quezon City Government*.

21st Quezon City Council. 2020c. SP-2983, S-2020:An Ordinance Prohibiting The Indiscriminate And Improper Disposal Of Face Masks And Other COVID-19 Protective Gears And Products In All Places Within The Territorial Jurisdiction Of Quezon City And Prescribing Penalties For Any Violation Thereof. *Quezon City Government*.

21st Quezon City Council. 2020d. SP-2966, S-2020: An Ordinance Approving Supplemental Budget Number 5, General Fund For Fiscal Year 2020, In The Amount Of One Billion One Hundred Fifty-five Million Five Thousand And Eight Hundred One Pesos (PHP1,155,005,801.00), To Fund Various Maintenance And Other Operating Expenses, Property, Plant And Equipment Expenditures In Response To COVID-19 Pandemic And For Other Purposes. *Quezon City Government*.

21st Quezon City Council. 2020e. SP-2958, S-2020: An Ordinance Establishing Individual Quarantine Protocols During The COVID-19 Pandemic. *Quezon City Government*.

21st Quezon City Council. 2020f. SP-2959, S-2020: An Ordinance Providing Guidelines For Special Concern Lockdown To Contain The COVID-19 Pandemic. *Quezon City Government*.

21st Quezon City Council. 2020g. SP-2950, S-2020: An Ordinance Imposing A Kalinga Fare For All Tricycles And Pedicab-for-hire In Quezon City During The Community Quarantine Due To COVID-19 Pandemic. *Quezon City Government*.

21st Quezon City Council. 2020h. SP-2947, S-2020: An Ordinance Amending Ordinance No. SP-2905, S-2020 Entitled ‘AN ORDINANCE SETTING PUBLIC SAFETY HOURS WITHIN THE TERRITORIAL JURISDICTION OF QUEZON CITY FROM 8:00 P.M. TO 5:00 A.M. DUE TO THE CORONAVIRUS DISEASE 2019 (COVID-19) PANDEMIC, PROVIDING PENALTIES FOR VIOLATION THEREOF, AND FOR OTHER PURPOSES’, By Setting An New Public Safety Hours Commencing From 10:00 PM To 5:00 AM. *Quezon City Government*.

21st Quezon City Council. 2020i. SP-2943, S-2020: An Ordinance Enabling COVID-19 Testing Of Select At-risk Quezon City Barangay Frontliners And Barangay Health Workers Who, In The Course Of Performing Their Duties, May Have Prolonged Close Contact With A Suspect, Confirmed Or Probable COVID-19 Case Within The Jurisdiction Of Quezon City. *Quezon City Government*.

21st Quezon City Council. 2020j. SP-2935, S-2020: An Ordinance Requiring The Mandatory COVID-19 Testing For All Persons Deprived Of Liberty (PDL) Before Admission To The Quezon City Jail And Other Similar Circumstances. *Quezon City Government*.

21st Quezon City Council. 2020k. SP-2926, S-2020: An Ordinance Granting Hazard Pay Of Two Hundred Pesos (PPH200.00) Per Day To All Barangay Personnel Who Physically Reported For Work In The Implementation Of The Enhanced Community Quarantine (ECQ) In Their Barangay Relative To The Covid-19 Pandemic. *Quezon City Government*.

21st Quezon City Council. 2020l. SP-2922, S-2020:An Ordinance Approving The Supplemental Budget No. 3, General Fund For Fiscal Year 2020, In The Amount Of One Billion And Four Hundred Thirty Million Pesos (PHP1,430,000.00) To Fund Various Maintenance And Other Operating Expenses In Response To COVID-19 Pandemic And For Other Purposes. *Quezon City Government*.

21st Quezon City Council. 2020m. SP-2923, S-2020:An Ordinance Granting Hazard Pay To The Quezon City Government Personnel Who Physically Report For Work During The Period Of Implementation Of Enhanced Community Quarantine Relative To The COVID-19 Pandemic. *Quezon City Government*.

21st Quezon City Council. 2020n. SP-2924, S-2020: An Ordinance Granting Of Special Risk Allowance To The Frontline Quezon City Public Health Workers During The Period Of Enhanced Community Quarantine Relative To The COVID-19 Pandemic. *Quezon City Government*.

21st Quezon City Council. 2020o. SP-2909, S-2020:An Ordinance Approving The Supplemental Budget No. 1, General Fund For The Fiscal Year 2020, In The Amount Of Two Billion And Eight Hundred Million Pesos (PHP2,800,000.00), In Response To COVID-19 Pandemic And For Other Operational Expenses. *Quezon City Government*.

21st Quezon City Council. 2020p. SP-2911, S-2020:An Ordinance Prohibiting Any Person, Whether Natural Or Juridical, From Committing Any Act Or Forms Of Discrimination Against Any Corona Virus (COVID-19) Infected Persons, Recovered Patients, Persons Under Monitoring (PUMs) Or Persons Under Investigation (PUIs), And Against Public And Private Doctors, Nurses, Health Workers, Emergency Personnel And Volunteers, And Other Service Workers, Who Are Assigned In Hospitals And/or Other Health Care Institutions / Centers Where COVID-19 Patients Are Being Treated Situated Within The Territorial Jurisdiction Of Quezon City, And Providing Penalties For Violations Therefor. *Quezon City Government*.

21st Quezon City Council. 2020q. SP-2912, S-2020:An Ordinance Granting Financial Assistance To All Frontliners Who Tested Positive To Corana Virus Disease 2019 (COVID-19), And Appropriating Funds Therefor. *Quezon City Government*.

21st Quezon City Council. 2020r. SP-2907, S-2020:An Ordinance Establishing Protocols For The Management Of The Deceased During The COVID-19 Pandemic. *Quezon City Government*.

21st Quezon City Council. 2020s. SP-2905, S-2020:An Ordinance Setting Public Safety Hours Within The Territorial Jurisdiction Quezon City From 8:00PM To 5:00AM, Due To The Corona Virus Disease 2019 (COVID-19) Pandemic, Providing Penalties For Violation Therefor, And For Other Purposes. *Quezon City Government*.

21st Quezon City Council. 2021a. SP-3082, S-2021: An Ordinance Suspending The Imposition Of Penalties, Interests And Surcharges To Late Payments Of Tricycle Franchise Renewal During Community Quarantine Only Due To COVID-19 Outbreak, Subject To Existing Laws, Rules And Regulations. *Quezon City Government*.

21st Quezon City Council. 2021b. SP-3061, S-2021: An Ordinance Amending Ordinance No. SP-2905, S-2020, Entitled ‘AN ORDINANCE SETTING PUBLIC SAFETY HOURS WITHIN THE TERRITORIAL JURISDICTION OF QUEZON CITY FROM 8:00PM TO 5:00AM DUE TO THE CORONAVIRUS DISEASE 2019 (COVID-19) PANDEMIC, PROVIDING PENALTIES FOR VIOLATION THEREOF, AND OR OTHER PURPOSES’, For The Purpose Of Suspending The Enforcement Of The Same And Giving Authority To The City Mayor To Reimpose The Same When Necessary To Respond To A Surge In COVID-19 Cases. *Quezon City Government*.

21st Quezon City Council. 2021c. SP-3048, S-2021: An Ordinance Regulating Covid-19 Vaccination Sites And Programs In Quezon City. *Quezon City Government*.

21st Quezon City Council. 2021d. SP-3032, S-2021:An Ordinance Prohibiting The Unauthorized Or Fraudulent Sale, Distribution Or Administration Of COVID-19 Vaccination Cards, And Other Frauds In Relation To COVID-19 Vaccination And Providing Penalties For Violation Thereof And For Other Purposes. *Quezon City Government*.

21st Quezon City Council. 2021e. SP-3029, S-2021:An Ordinance Granting Financial Assistance To All Frontliners Who Are Tested Positive To CORONAVIRUS DISEASE 2019 (COVID-19), And Appropriating Funds Therefor. *Quezon City Government*.

21st Quezon City Council. 2021f. SP-3031, S-2021:An Ordinance Prohibiting The Selling And Buying Of Government Procured COVID-19 Vaccine And Selling And Buying Of Slots To Avail Of COVID Vaccination And Providing Penalties For Violation Thereof. *Quezon City Government*.

21st Quezon City Council. 2021g. SP-3024, S-2021:An Ordinance Granting Daily Allowance To The COVID-19 Vaccination Volunteer Medical Staff. *Quezon City Government*.

21st Quezon City Council. 2021h. SP-3014, S-2021:An Ordinance Giving Authority To The City Mayor To Issue Emergency Measures To Regulate The Sale, Distribution, And Consumption Of Liquor And Other Intoxicating Beverages Within The City, Enforceable During The Period Of Public Health Emergency Due To The COVID-19 Pandemic, Subject To The Inter-Agency Task Force For The Management Of Emerging Infectious Diseases (IATF-MEID) And Metro Manila Council Guidelines. *Quezon City Government*.

21st Quezon City Council. 2021i. SP-3003, S-2021:An Ordinance Further Amending Ordinance No. SP-2905, S-2020, Entitled ‘An Ordinance Setting Public Safety Hours Within The Territorial Jurisdiction Of Quezon City From 8:00 P.M. To 5:00 A.M. Due To The CORONAVIRUS DISEASE 2019 (COVID-19) Pandemic, Providing Penalties For Violation Thereof, And For Other Purposes,’ By Setting A New Public Safety Hours Commencing From 12:00 A.M. To 4:00 A.M. *Quezon City Government*.

21st Quezon City Council. 2022a. SP-3125, S-2022: An Ordinance Amending Subparagraph B (3) Of Paragraph A And Subparagraph B (3) Of Paragraph B, Both Under Section 3 Of Ordinance No. 3114, S-2022, Entitled ‘AN ORDINANCE REVISING ORDINANCE NO. SP-2273, S-2014, ENTITLED AND ORDINANCE ADOPTING THE SEAL OF GOOD HOUSEKEEPING IN ALL BARANGAYS OF QUEZON CITY, CONSOLIDATING THE AMENDMENTS PROVIDED IN ORDINANCE NOS. SP-2523, S-2016, SP-2588, S-2017, AND SP-2898, S-2020 AND INCORPORATING INDICATORS FOR COVID-19 REPOSPONSE.’ *Quezon City Government*.

21st Quezon City Council. 2022b. SP-3114, S-2022: An Ordinance Revising Ordinance No. SP-2273, S-2014, Entitled ‘AN ORDINANCE ADOPTING THE SEAL OF GOOD HOUSEKEEPING IN ALL BARANGAYS OF QUEZON CITY,’ Consolidating Amendments Provided In Ordinance Nos. SP-2523, S-2016, SP-2588, S-2017, And SP-2898, S-2020 And Incorporating Indicators For COVID-19 Response. *Quezon City Government*.

21st Quezon City Council. 2022c. SP-3105, S-2022: An Ordinance Amending Ordinance No. SP-2987, S-2020, Entitled ‘AN ORDINANCE MANDATING STRICT OBSERVANCE OF MINIMUM HEALTH PROTOCOLS BY PUBLIC TRANSPORTATION DURING THE COVID-19 PANDEMIC’ In Light Of The Declaration Of Alert Level 1 In The National Capital Region. *Quezon City Government*.

21st Quezon City Council. 2022d. SP-3102, S-2022: An Ordinance Suspending The Implementation Of Ordinance No. SP-2985, S-2020, Entitled ‘AN ORDINANCE PROVIDING FOR A SPECIAL PROTECTION OF CHILDREN AGAINST CORONAVIRUS DISEASE 2019 (COVID-19) BY SETTING CHILDREN PROTECTION HOURS WITHIN THE TERRITORIAL JURISDICTION OF QUEZON CITY, PROVIDING PENALTIES FOR VILOATION THEREOF, AND FOR OTHER PURPOSES’ In Light Of The Decalaration Of Alert Level 1 In Metro Manila, And Granting Authority To The City Mayor To Restore The Enforcement Of The Same In Accordance With The National Government Policies And Health Exigency. *Quezon City Government*.

21st Quezon City Council. 2022e. SP-3101, S-2022: An Ordinance Suspending The Implementation Of Ordinance No. SP-2907, S-2020, As Amended By Ordinance No. SP-3080, S-2022, Entitled ‘AN ORDINANCE ESTABLISHING PROTOCOLS FOR THE MANAGEMENT OF THE DECEASED DURING THE COVID-19 PANDEMIC’. *Quezon City Government*.

21st Quezon City Council. 2022f. SP-3089, S-2022 An Ordinance Suspending Ordinance No. Sp-3076, S-2022, Entitled "An Ordinance Regulating The Mobility Of Individuals Unvaccinated For Covid-19 In Quezon City In Light Of The Declaration Of Alert Level 2 In Metro Manila, And Granting Authority To The City Mayor To Restore The Enforcement Of The Same In Accordance With The National Government Issuances. *Quezon City Government*.

21st Quezon City Council. 2022g. SP-3077, S-2022: An Ordinance Amending Ordinance No. SP-3029, S-2021, Entitled ‘AN ORDINANCE GRANTING FINANCIAL ASSISTANCE TO ALL FRONTLINERS WHO ARE TESTED POSITIVE TO CORONAVIRUS DISEASE 2019 (COVID-19) AND APPROPRIATING FUNDS THEREFOR’, To Limit The Ten Thousand Pesos (PHP10,000.00) Benefit Only To Cases Requiring Hospitalization. *Quezon City Government*.

21st Quezon City Council. 2022h. SP-3076, S-2022: An Ordinance Regulating The Mobility Of Individuals Unvaccinated For COVID-19 In Quezon City. *Quezon City Government*.

21st Quezon City Council. 2022i. SP-3080, S-2022: An Ordinance Amending Ordinance No. SP-2907, S-2020, Entitled ‘AN ORDINANCE ESTABLISHING PROTOCOLS FOR THE MANAGEMENT OF THE DECEASED DURING THE COVID-19 PANDEMIC’ To Allow Limited Wakes Or Burol. *Quezon City Government*.

21st Quezon City Council. 2022j. SP-3078, S-2022: An Ordinance Amending Ordinance No. SP-2958, S-2020, Entitled ‘AN ORDINANCE ESTABLISHING INDIVIDUAL QUARANTINE PROTOCOLS DURING THE COVID-19 PANDEMIC’, As Amended By Ordinance No. SP-3034, S-2021. *Quezon City Government*.
